# Nutritional Status of Adolescents in Eastern Sudan: A Cross-Sectional Community-Based Study

**DOI:** 10.3390/nu16121936

**Published:** 2024-06-19

**Authors:** Mohammed Ahmed A. Ahmed, Abdullah Al-Nafeesah, Jaber Alfaifi, Ashwaq AlEed, Ishag Adam

**Affiliations:** 1 Faculty of Medicine, Gadarif University, Gadarif 32211, Sudan; 2 Department of Pediatrics, College of Medicine, Qassim University, Buraydah 52571, Saudi Arabia; a.alnafeesah@qu.edu.sa; 3 Department of Child Health, College of Medicine, University of Bisha, Bisha 61922, Saudi Arabia; jalfaifi@ub.edu.sa; 4 Department of Obstetrics and Gynecology, College of Medicine Qassim University, Buraydah 52571, Saudi Arabia; ishagadam@hotmail.com

**Keywords:** adolescent, age, thinness, stunting, overweight, obesity

## Abstract

Background: Malnutrition among adolescents is a major public health issue. This problem is particularly pressing in Sudan, an African country where there is scarce published data on the nutritional status of adolescents. In this study, we aimed to assess the nutritional status of adolescents in eastern Sudan. Methods: A community-based cross-sectional survey was carried out in Gadarif, eastern Sudan. A questionnaire was used to collect sociodemographic data, and the anthropometric measurements (weight and height) of adolescent participants were recorded. Height-for-age and body mass index-for-age Z-scores were calculated using the WHO anthropometric standards. Binary and multivariate multinomial regression analyses were performed. Results: A total of 388 adolescents were included in this survey, 207 (53.4%) were female, and 181 (46.6%) were male. The median (interquartile) age was 13.9 (12.0–16.0) years. The results showed that a total of 29 (7.5%), 93 (24.0%), 33 (8.5%), and 16 (4.1%) adolescents were stunted, thin, overweight, and obese, respectively. None of the investigated factors (age, sex, parents’ education levels, and occupation) were associated with stunting. In the multivariate multinomial analysis, the male sex was associated with thinness (OR = 2.41, 95.0% CI = 1.47–3.94). Moreover, adolescents whose mothers had an education lower than secondary level were at a lower risk of overweight/obesity (OR = 0. 0.35, 95.0% CI = 0. 0.35). Conclusions: While both undernutrition and overnutrition exist in eastern Sudan, undernutrition is more common. Male sex and mothers’ education levels are associated with malnutrition.

## 1. Introduction

Adolescence is the period of human age between 10 and 19 years. This period represents a pivotal phase of puberty, marked by significant physical, mental, and psychosocial transformations necessitating a diverse array of nutrients [1]. The global population of adolescents is about 16% or 1.3 billion [2]. Malnutrition during adolescence is associated with long-term consequences, including potential reductions in cognitive function, school performance, and reproductive life [1,3,4].

A recent report revealed the increasing burden of both underweight and obesity among children and adolescents worldwide, particularly in low- and middle-income countries [5]. Moreover, the double burden of malnutrition (stunting and overweight/obesity) is widely prevalent among adolescents in Africa [6]. Several studies have been published on the prevalence of nutritional status and its associated factors among adolescents in sub-Saharan Africa [6,7,8,9,10,11,12,13,14,15,16,17,18]. For example, different rates of stunting among adolescents were reported in various African countries [6,7,10,11,14,15,19]. The association between stunting and several factors, such as age [20,21] and sex [7,15,17], and the varying prevalence of thinness among adolescents in different African countries [8,10,11,14,19] have also been reported. Aside from stunting and thinness, different rates of overweight and obesity have been observed in several African countries [8,9,11,22]. 

However, there are few published studies on the prevalence of nutritional status and its associated factors among adolescents in Sudan, the third-largest African country [20,23]. In 2019, 4.3% of girls and 8.7% of boys categorized as children and adolescents (5–19 years) in Sudan were reported to exhibit thinness, indicating that Sudan has one of the largest numbers of malnourished children and adolescents in the world [24]. Malnutrition is one of the main health problems among children in our selected study area of Gadarif City, especially among children whose mothers had low educational levels and in children living with large families [25]. Moreover, malnutrition is one of the main causes of pediatric admission and death in Gadarif Hospital in eastern Sudan [26]. Furthermore, a community-based survey showed that 15.6% of the adolescents in New Halfa in eastern Sudan were underweight [27]. On 15 April 2023, the Sudanese military and the Rapid Support Force in Khartoum State commenced an armed conflict. The war eventually extended to other regions of Sudan, leading to at least 50,000 deaths, 2.6 million internal displacements, and 730,000 forced to seek refuge elsewhere [28]. 

Providing data on the prevalence and associated factors for undernutrition in adolescents in Sudan is necessary for academicians, health planners, and providers to facilitate the planning of services and food assistance and to help manage the negative impact of undernutrition on future adults in Sudan. Therefore, this study aimed to assess the nutritional status of adolescents in eastern Sudan.

## 2. Materials and Methods

### 2.1. Study Design and Setting

A community-based cross-sectional study was conducted in Gadarif City, eastern Sudan, from August to October 2023. Gadarif City was chosen because it might be representative of the other regions of Sudan, and a previous study has shown a high rate of malnutrition among adults in this region [29]. Gadarif City, the capital of Gadarif State, which is on the Ethiopian border, has a total population of 1,400,000 [30]. The Gadarif locality (including Gadarif City) is the largest of the 11 localities in Gadarif State. This study adhered to the Strengthening the Reporting of Observational Studies in Epidemiology (STROBE) guidelines [31].

### 2.2. Sampling

Gadarif City is divided into four squares (Mouraba) comprising 13 blocks (Hay). The total population in each block was obtained from the local authorities. The desired sample size was 388 adolescents, including both males and females. The probability proportion, including the number of adolescents enrolled from each block, was proportionate to the total number of adolescents in each block. 

### 2.3. Inclusion and Exclusion Criteria

The inclusion criteria included adolescents aged between 10 and 19 residing in the study area of Gadarif City. The exclusion criteria consisted of those younger than 10 or older than 19, those who did not consent to participate in the study component on diet, and those who were sick, pregnant, or lactating.

### 2.4. Study Variables and Measures

After the adolescents and their guardians signed the informed consent and witness form, two trained medical officers conducted face-to-face interviews with the participants and completed the study questionnaire. The questionnaire included data on date of birth (confirmed via ID to calculate the participant’s age), sex (male or female), parents’ educational levels (<secondary or ≥secondary), mother’s occupation (housewife or employed), and father’s occupational status (laborer or skilled worker).

Following the interview process, each participant was weighed. After removing shoes and excess clothing, the adolescents were instructed to stand still with their hands by their sides on the weighing scale. Their weights and heights were measured twice and recorded, and then the means of the two measurements for weight and height were computed and documented as the final values. A Seca 874 weighing scale (Seca, Deutschland, Germany) was used to weigh each adolescent (to the nearest 100 g), and its well-calibrated scales were adjusted to zero before each measurement. A Seca stadiometer (Shorr Board, Olney, MD, USA) was used to measure the adolescents’ height to the nearest 0.1 cm. During the measurement, the adolescents stood straight with their backs against a wall and feet together. 

The WHO reference was used to compute the body mass index-for-age Z-score (BMIZ) and height-for-age Z-score (HAZ) [32]. Stunting was considered for a HAZ of <−2 SD. Thinness was considered when the BMIZ was <−2 SD. Overweight was considered when 1 SD < BMIZ ≤ 2 SD, while obesity was considered when BMIZ > 2 SD.

### 2.5. Sample Size

The sample size (388 adolescents) was calculated using the OpenEpi Menu [33]. The sample size of 388 adolescents (*n*) was estimated based on an assumed prevalence (25.0%) of thinness among adolescents (ratio of 3:1). This assumption (25.0% of the adolescents would have thinness) was based on the recently reported prevalence of thinness among adolescents in north Sudan [23]. We then assumed that 60.0% of thin adolescents were female, while 40.0% of the adolescents with normal weight were female. This sample size (388) had a confidence interval (CI) of 95% and a precision of 0.05. 

### 2.6. Statistics

Analysis was performed using the Statistical Package for the Social Sciences^®^ for Windows Version 22.0 (SPSS Inc., New York, NY, USA). The age, weight, height, HAZ, and BMIZ values of the adolescents were continuous data found to be non-normally distributed after the Shapiro–Wilk test evaluation. Therefore, these data were expressed as the median (interquartile range (IQR)) and compared between two groups using a non-parametric test (the Mann–Whitney U test). A chi-square test was used to compare categorized variables expressed as a frequency (proportion). Normal weight, thinness, and overweight/obese were used as dependent variables for univariate multinomial analysis, while stunting was used as the dependent variable for univariate binary analysis. The gathered sociodemographic data, namely the adolescents’ age, gender, and their parents’ educational levels, were used as the independent variables for both analyses. Thereafter, variables with *p* < 0.20 in the univariate analysis were shifted to perform multivariate regression with backward elimination. The results of adjusted odds ratios (AORs) and a 95% CI were calculated, and a *p*-value of <0.05 was considered statistically significant. 

## 3. Results

A total of 388 adolescents were enrolled in this study, 207 (53.4%) were female, and 181 (46.6%) were male. The median (interquartile (IQR)) age was 13.9 (12.0–16.0) years. Of the 388 adolescents, 271 (69.8%) mothers and 291(75.0%) fathers had secondary school or higher education levels, while 315 (81.2) mothers were housewives (Table 1). Six (1.5%) adolescents were smokers, and three (0.8%) were users of tobacco snuff.

### 3.1. Anthropometric Measurements

The median (IQR) height of the enrolled adolescents in this study was 156.0 (146.5–164.0) cm, while the median (IQR) of their weight was 42.7 (34.0–51.7) kg. There was no significant difference in the median (IQR) height (157.0 [146.0–168.0] cm versus 156.0 [148.0–162.1] cm, *p* = 0.114) or weight (41.6 [32.8–51.0] kg versus 43.3 [35.7–53.0] kg, *p* = 0.122) between males and females. The adolescents’ median (IQR) HAZ and BMIZ values were −0.42 (−1.1 to 0.34) and −0.96 (−1.9 to 0.26), respectively.

### 3.2. Factors Associated with Stunting

Twenty-nine (7.5%) adolescents had stunting. The investigated factors (age, sex, parents’ education, and occupation) were not associated with stunting (Table 1). 

### 3.3. Factors Associated with Thinness, Overweight/Obesity

A total of 93 (24.0%), 246 (63.4%), 33 (8.5%), and 16 (4.1%) adolescents were thin, normal weight, overweight, and obese, respectively. There were no significant differences in age, parents’ education, father’s education, and smoking in adolescents with normal weight, thinness, overweight, and obesity. However, significantly fewer females were among the thin adolescents (Table 2). For further analysis, we added the overweight and obese as one group (overweight/obese, n = 49).

In the univariate multinomial analysis, the male sex was associated with thinness (OR = 2.37, 95.0% CI = 1.45–3.88), whereas age, parents’ education, and occupation were not associated with thinness. 

Moreover, adolescents whose mothers had an education lower than secondary level were at a lower risk of overweight/obesity (OR = 0.35, 95.0% CI = 0.15–0.81). Age, sex, parents’ occupation, and paternal education were not associated with overweight/obesity (Table 3).

In the multivariate multinomial analysis, the male sex was associated with thinness (OR = 2.41, 95.0% CI = 1.47–3.94). Moreover, adolescents whose mothers had an education lower than secondary level were at a lower risk of overweight/obesity (OR = 0. 0.35, 95.0% CI = 0. 0.35) (Table 4).

## 4. Discussion

The main findings of this study revealed that 7.5%, 24.0%, and 12.6% of the adolescents were stunted, thin, and overweight/obese, respectively. The observed prevalence (7.5%) of stunting among adolescents in this study was lower than the prevalence of stunting that we recently reported among adolescents in north Sudan (19.8%) [23]; among schoolchildren aged 5–15 years in eastern Sudan (22.1%) [20]; among school-age children in Ebonyi State in Nigeria (9.9%) [19]; among street adolescents in the city of Adama, Oromia Region, Ethiopia (34.1%) [10], and Tanzania (18%) [14]; and among 7625 adolescents from 8 countries in sub-Saharan Africa (18.1%) [15]. Moreover, our results revealed a lower prevalence of stunting compared with the pooled prevalence of stunting reported in a meta-analysis among adolescents in South Africa (9·6%) [6] and Ethiopia (22.4%) [7]. On the other hand, the prevalence (7.5%) of stunting in our study was higher than that recently reported (5.7%) among school adolescents in high schools in the town of Durame in Ethiopia [11]. Although none of the investigated factors (age, sex, parents’ education, and occupation) were associated with stunting in our study, several previous studies in Africa have shown that increasing age [20,21], male sex [7,15], and female sex [17] were associated with stunting. The difference in the prevalence of stunting between our study and the other studies could be attributed to the differences in sociodemographic, economic, and genetic factors in the different regions. A previous study in Southeast Asia showed that several socio-economic and demographic factors, such as age, sex, father’s occupation, and family income, were associated with stunting among adolescents [34]. 

In the current study, 24.0% of the adolescents were thin, and the male sex was associated with thinness (OR = 2.41). The prevalence of thinness in our study was higher than what was reported (pooled prevalence of 22.0%) in a recent meta-analysis, which included 39 articles and enrolled 24,716 schoolchildren in neighboring Ethiopia [21], as well as among 10–17-year-old adolescents in Rivers State University, Nigeria (6.9%) [8]; in Ebonyi State, Nigeria (7.2%) [19]; and in Tanzania (14%) [14]. However, the observed prevalence of thinness was lower than the prevalence of thinness (32.3%) recently reported among schoolchildren aged 5–15 years in eastern Sudan [20]. 

In the present study, as well as in previous studies in north Sudan [8], eastern Sudan [20], Ethiopia [5], and Nigeria [19], thinness was associated with the male sex. Specifically, in this study, the male sex was associated with a 2.41 higher risk for thinness. Some factors could explain the difference in the association of thinness between adolescent boys and girls. For example, the difference in age of maturation between boys and girls could explain the increased susceptibility to thinness observed between them [15,35]. The traditional practice of allowing boys to spend more time engaged in outdoor activities and physical pursuits that require more energy/nutrients could also contribute to their thinner physiques compared with girls. By contrast, girls may spend more time indoors, leading to higher food intake [36]. In addition, some cultural beliefs encourage girls to be overweight or obese, associating such physiques with beauty, attractiveness, and wealth [37].

In the current study, 8.5% and 4.1% of the adolescents were overweight and obese. Our results were slightly lower than the prevalence of overweight and obesity reported in sub-Saharan Africa. For example, of the 498 adolescents in Ethiopia, 9.2% were overweight [11], and in the Bauchi metropolis, Nigeria, 11.0% and 9.7% of the adolescents were overweight and obese, respectively [22]. Moreover, at Rivers State University in Nigeria, 19.9% of 10–17-year-old adolescents were overweight/obese [8]. However, the prevalence of overweight and obesity observed in the current study was higher than the prevalence of overweight (0.5%) and obesity (2.3%) recently reported in north Sudan [23].

In the current study, adolescents whose mothers had an education lower than secondary level were at a lower risk of overweight/obesity (OR = 0.0.35), which means that the increased prevalence of overweight/obesity was associated with the mothers’ education levels. This finding aligns with the results of a recent meta-analysis (2024) that included 35 studies, showing that higher maternal education was associated with the weight-for-age Z-score (WAZ) and HAZ [3]. On the other hand, previous studies in north Sudan [22] and eastern Sudan [20] reported that maternal education level had no significant effect on nutritional status [20]. Several previous studies in different regions in Africa presented different results of parental education on adolescent nutrition [38,39,40]. Educated parents could have a positive influence on the nutrition of their children (adolescents) through their ability to provide the optimum environmental and nutritional status of the households [41]. On the other hand, education is associated with family income. Income is an important determinant of food availability (and type of food available) and nutritional status [34]. Although our study showed no difference in the prevalence of overweight/obesity between males and females, previous studies have shown sex differences in the prevalence of overweight/obesity among adolescents [36,42]. The difference in the prevalence of overweight/obesity between male and female adolescents was attributed to the difference in types of consumed food, eating habits, and physical activities [36,42]. 

It is worth mentioning that weight, height, and associated BMIZ values were used to determine the nutritional status of adolescents in this study. The BMI is not without limitations when assessing the status of undernutrition among children and adolescents. Previous research has shown some limitations of using BMI to assess nutritional status, such as studies in edema, inappropriate muscle mass in cerebral palsy, and debilitating diseases (cancer) [43,44].

## 5. Conclusions

The results of the community-based cross-sectional survey conducted in this study determined that while undernutrition and overnutrition exist in eastern Sudan, undernutrition is more common. Moreover, our study results showed that male sex and mothers’ education levels are associated with malnutrition.

Therefore, there is a need for intervention programs that consider the dissemination of nutrition knowledge at the population level to ameliorate the nutritional status among adolescents; however, the implementation of such intervention programs could be difficult due to the ongoing war in Sudan.

## Figures and Tables

**Table 1:** Univariate analysis of the factors associated with stunting among adolescent schoolchildren in eastern Sudan, 2023.

Variables		Total (n = 388)	Adolescents with Stunting (n = 29)	Adolescents without Stunting (n = 359)	OR	95% CI	*p*
		Median (interquartile range)					
Age, years		13.9 (12.0–16.0)	13.1 (11.3–15.7)	14.0 (12.1–16.0)	0.91	0.78–1.06	0.260
		Frequency (proportion)					
Sex	Male	181 (46.6)	15 (51.7)	192 (53.5)	0.51	0.12–2.19	0.518
	Female	207 (53.4)	14 (48.3)	167 (46.5)		Reference	
Mother’s education	≥Secondary level	271 (69.8)	22 (75.9)	249 (69.4)		Reference	
	˂Secondary level	117 (30.2)	7 (24.1)	110 (30.6)	0.72	0.29–1.73	0.465
Mother’s occupation	Housewife	315 (81.2)	23 (79.3)	292 (81.3)		Reference	
	Employed	73 (18.8)	6 (20.7)	67 (18.7)	1.13	0.44–2.90	0.788
Father’s education	≥Secondary level	291 (75.0)	22 (75.9)	269 (74.9)		Reference	
	˂Secondary level	97 (25.0)	7 (24.1)	90 (25.1)	0.54	0.12–3.33	0.416
Father’s occupation	Labor	236 (60.8)	15 (51.7)	221 (61.6)		Reference	
	Skilled	152 (39.2)	14 (48.3)	138 (38.4)	1.56	0.38–6.36	0.529

**Table 2:** Comparison of sociodemographic characteristics between adolescents with thinness, overweight, and obesity in eastern Sudan, 2023.

Variable		Normal Weight (n = 246)	Thinness (n = 93)	Overweight (n = 33)	Obese (n = 16)	*p*
		Median (interquartile range)				
Age, years		13.7 (11.7–16.0)	13.8 (12.3–15.9)	14.9 (13.1–16.7)	16.7 (11.8–16.2)	0.681
		Frequency (proportion)				
Sex	Male	101 (41.1)	58 (62.4)	14 (42.4)	8 (50.0)	0.006
	Female	145 (58.9)	35 (37.6)	19 (57.6)	8 (50.0)	
Mother’s education	≥Secondary level	167 (67.9)	62 (66.7)	28 (84.8)	14 (87.5)	0.079
	˂Secondary level	79 (32.1)	31 (33.3)	5 (15.2)	2 (12.5)	
Mother’s occupation	Housewife	205 (83.3)	73 (78.5)	28 (84.8)	9 (56.2)	0.046
	Employed	41 (16.7)	20 (21.5)	5 (15.2)	7 (43.8)	
Father’s education	≥Secondary level	180 (73.2)	70 (75.3)	26 (78.8)	15 (93.8)	0.296
	˂Secondary level	66 (26.8)	23 (24.7)	7 (21.2)	1 (6.3)	
Father’s occupation	Labor	146 (59.3)	63 (67.7)	14 (42.4)	8 (50.0)	0.388
	Skilled	100 (40.7)	30 (32.3)	19 (57.6)	8 (50.0)	
Smoking/snuff	No	241 (98.0)	90 (96.8)	33 (100)	16 (100)	0.647
	Yes	5 (2.0)	3 (3.2)	0 (0)	0 (0)	

**Table 3:** Univariate multinomial analysis of factors associated with thinness and overweight/obesity among adolescents in eastern Sudan, 2023.

Variable		Underweight/Thinness		Overweight/Obese	
		Odds Ratios (95.0% Confidence Interval)	*p*	Odds Ratios (95.0% Confidence Interval)	*p*
Age, years		1.03 (0.93–1.13)	0.527	1.11 (0.98–1.25)	0.098
Sex	Male	2.37 (1.45–3.88)	<0.001	1.17 (0.63–2.16)	0.619
	Female	Reference		Reference	
Mother’s education	≥Secondary level	Reference		Reference	0.015
	˂Secondary level	1.05 (0.63–1.75)	0.831	0.35 (0.15–0.81)	
Mother’s occupation	Housewife	Reference		Reference	0.196
	Employed	0.73 (0.40–1.32)	0.302	0.61 (0.29–1.28)	
Father’s education	≥Secondary level	Reference		Reference	0.126
	˂Secondary level	0.89 (0.51–1.55)	0.695	0.53 (0.23–1.19)	
Father’s occupation	Labor	1.43 (0.86–2.38)	0.157	0.84 (0.45–1.55)	0.582
	Skilled	Reference		Reference	

**Table 4:** Multivariate multinomial analysis of factors associated with thinness and overweight/obesity among adolescents in eastern Sudan, 2023.

Variable		Underweight/Thinness		Overweight/Obesity	
		Odds Ratios (95.0% Confidence Interval)	*p*	Odds Ratios (95.0% Confidence Interval)	*p*
Sex	Male	2.41 (1.47–3.94)	<0.001	1.08 (0.58–2.03)	0.789
	Female	Reference		Reference	
Mother’s education	≥Secondary level	Reference		Reference	0.017
	˂Secondary level	1.15 (0.68–1.94)	0.585	0.35 (0.15–0.82)	

## Data Availability

The original contributions presented in the study are included in the article, further inquiries can be directed to the corresponding author.
